# Dynamic control of tumor vasculature improves antitumor responses in a regional model of melanoma

**DOI:** 10.1038/s41598-020-70233-5

**Published:** 2020-08-06

**Authors:** Emmanuel M. Gabriel, Minhyung Kim, Daniel T. Fisher, Colin Powers, Kristopher Attwood, Sanjay P. Bagaria, Keith L. Knutson, Joseph J. Skitzki

**Affiliations:** 1 Department of Surgery, Section of Surgical Oncology, Mayo Clinic Florida, 4500 San Pablo Road, Jacksonville, FL 32224 USA; 2 Department of Immunology, Roswell Park Comprehensive Cancer Center, Buffalo, NY USA; 3 Department of Biostatistics, Roswell Park Comprehensive Cancer Center, Buffalo, NY USA; 4 Department of Immunology, Mayo Clinic, Jacksonville, FL USA; 5 Department of Surgical Oncology, Roswell Park Comprehensive Cancer Center, Buffalo, NY USA

**Keywords:** Cancer therapy, Cancer imaging, Melanoma

## Abstract

Despite advances in therapy for melanoma, heterogeneous responses with limited durability represent a major gap in treatment outcomes. The purpose of this study was to determine whether alteration in tumor blood flow could augment drug delivery and improve antitumor responses in a regional model of melanoma. This approach to altering tumor blood flow was termed “dynamic control.” Dynamic control of tumor vessels in C57BL/6 mice bearing B16 melanoma was performed using volume expansion (saline bolus) followed by phenylephrine. Intravital microscopy (IVM) was used to observe changes directly in real time. Our approach restored blood flow in non-functional tumor vessels. It also resulted in increased chemotherapy (melphalan) activity, as measured by formation of DNA adducts. The combination of dynamic control and melphalan resulted in superior outcomes compared to melphalan alone (median time to event 40.0 vs 25.0 days, respectively, p = 0.041). Moreover, 25% (3/12) of the mice treated with the combination approach showed complete tumor response. Importantly, dynamic control plus melphalan did not result in increased adverse events. In summary, we showed that dynamic control was feasible, directly observable, and augmented antitumor responses in a regional model of melanoma. Early clinical trials to determine the translational feasibility of dynamic control are ongoing.

## Introduction

The incidence of melanoma has steadily risen in the United States since the 1970s, with approximately 96,000 cases estimated in 2019^1^. In-transit (IT) disease is an advanced presentation of melanoma and refers to metastases within the regional dermal and subdermal lymphatics between the primary melanoma and the draining lymph node basin^2^. Overall, approximately 4–11% of patients present with IT metastases^3,4^. Several factors are associated with an increased risk for IT disease, including thicker primary melanomas (> 1 mm), tumor ulceration, lymphovascular invasion (LVI), regional nodal involvement, and location of the primary on a lower extremity^5,6^.

A wide range of treatment options is available for IT melanoma, which is associated with poor outcomes even in the current, modern era^7,8^. Recent advances in immune checkpoint blockade, including anti-CTLA4 and anti-PD1 antibodies, as well as novel targeted therapies like Talimogene laherparepvec (T-VEC), have been brought to the forefront of treatment for patients with IT disease^8^. These systemic therapies are often used as first-line agents for IT disease and can be effective in a high proportion of patients. However, there still remain patients who do not obtain benefit from these systemic agents or who have contraindications to their use, such as a history of organ transplant, for example.

Prior to newer generation systemic therapies, a successful treatment for IT disease encompassed regional therapies, which consist of isolated limb perfusion (ILP) and isolated limb infusion (ILI)^9^. ILP involves vascular dissection of the inflow and outflow vessels of the involved extremity followed by delivery of high dose heated chemotherapy, most often including the agent melphalan, which causes DNA damage through alkylation and cross-linkage^10^. Though similar to ILP, ILI differs in that it involves percutaneous catheter based delivery of chemotherapeutic agents. Other technical differences between ILP and ILI include the perfusion temperature, oxygenation level, and length of treatment. ILP has achieved overall response rates as high as 80% and complete response rates of up to 55%^11,12^. While these response rates are favorable, they have been heterogeneous and often of limited durability. Thus, the development of novel, effective therapies is greatly needed in order to improve durable response rates for patients with IT melanoma.

One underdeveloped area of therapy for melanoma, including IT disease, is altering the tumor-associated vessels in order to optimize treatment delivery. The tumor vasculature serves as the interface between tumor and host. This vasculature is recognized as a critical factor that dictates the efficacy of all current systemically delivered cancer therapies, including chemotherapy as well as immunotherapy (for example effector lymphocyte trafficking and infiltration into tumor)^13,14^. The most successful modern chemotherapeutic agents require adequate distribution to the tumor via the circulation, or else they are rendered ineffective^15–17^. Several studies have characterized different mechanisms by which tumor vessels impede drug delivery^18–20^. These findings have had important implications on how best to overcome barriers to cancer therapy^21,22^. Alterations in tumor vessel flow and permeability have been shown to result in a significant impact on drug delivery or lymphocyte trafficking^23,24^. However, these studies have addressed either immune-mediated homing of effector cells to tumor or blockade of tumor cell neovascularization using anti-VEGF antibodies like bevacizumab^23,25^. The feasibility and effectiveness of altering blood flow to tumor, however, has not been well characterized, particularly with regard to enhancing systemically delivered therapies.

In the context of improving regional therapies for IT melanoma and based on the rationale that systemic drug delivery is dependent on the tumor vasculature, the purpose of this study was to develop a directly observable and reproducible method of optimal blood flow through tumor vessels that improves regional antitumor responses in a preclinical mouse model. Our method was termed “dynamic control” of the tumor vasculature. Our hypothesis was that optimal alteration of blood flow through tumor vessels would be feasible and would result in improved drug efficacy. The rationale for this hypothesis was that dynamic control would result in increased drug delivery to the tumor, thereby increasing exposure of the tumor to the drug and subsequently improving antitumor responses.

## Results

### Dynamic control with a saline bolus followed by phenylephrine (1) restores blood flow in non-functional tumor vessels and (2) transiently alters the blood flow in functional tumor vessels

**Figure 1:**
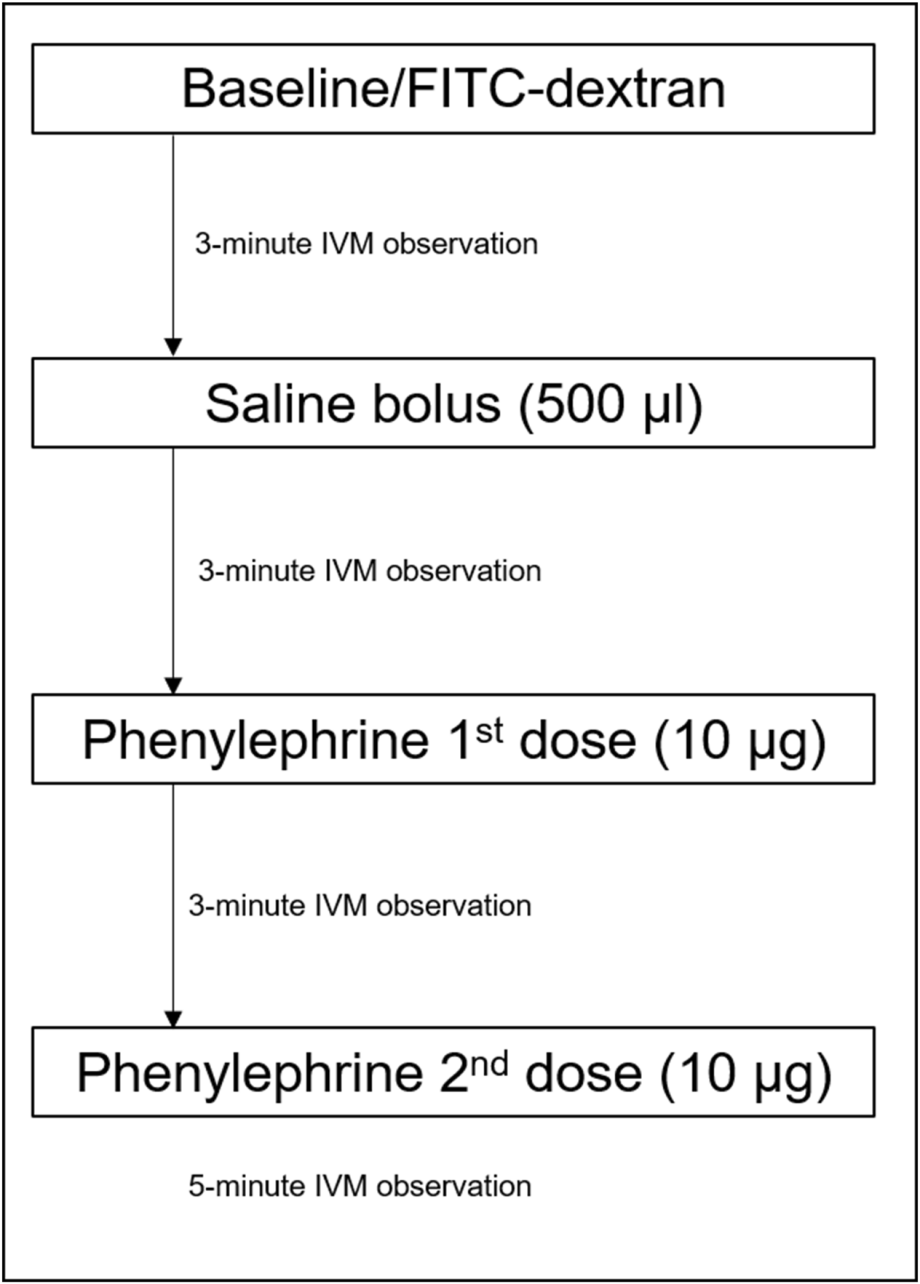
Protocol for the dynamic control of tumor vessels. Systemic volume expansion (500 μl PBS) followed by two doses of intravenous (tail vein) injection with phenylephrine (10 μg per dose) comprised the approach to dynamic control. Intravital microscopy (IVM) was used to perform real-time imaging of tumor-associated vessels, which was enhanced with FITC-dextran.

**Figure 2:**
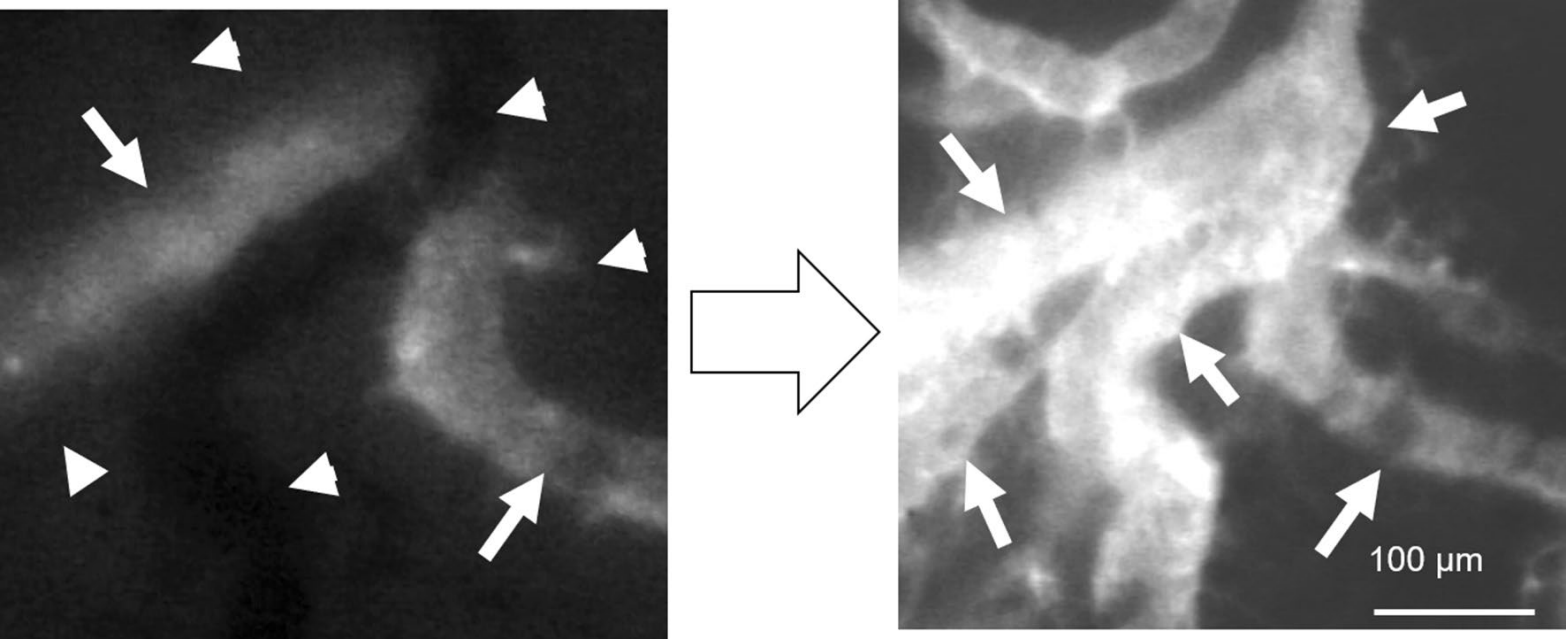
Restoration of blood flow through tumor-associated vessels using dynamic control. Left panel shows baseline IVM observation. The horizontally oriented vessel (arrows) demonstrated blood flow by the uptake of fluorescein. The vertically oriented vessel (arrowheads) did not demonstrate blood flow (absence of FITC-dex uptake). Right panel shows vessels after completion of the dynamic vessel control protocol.

The approach to dynamic control is shown in Fig. 1 and is further explained in the Methods section. In brief, for this set of experiments dynamic control consisted of an intravenous 500 μl saline bolus followed by two intravenous doses of phenylephrine (10 μg each) separated by 3 min. Figure 2 depicts how dynamic control optimized blood flow, using a colorectal cancer CT26 tumor-bearing BALB/c mouse as the example. On baseline imaging (left panel), a vertically oriented blood vessel (marked by arrowheads) did not demonstrate fluorescein isothiocyanate–dextran (FITC-dex) uptake, which was used as the intravital fluorescent dye. In contrast, there was a horizontally oriented vessel (marked by arrows) that did demonstrate FITC-dex uptake and was considered functional. Following dynamic control (right panel), the non-functional vertically oriented blood vessel had restored dye uptake. In addition, many other surrounding tumor-associated vessels not only demonstrated restored blood flow but also increased flow.

Supplemental Fig. 1 shows video examples of the results obtained from the dynamic control of tumor vessels using our protocol. Each video is shown in 2 × real-time speed and is marked with a date/time stamp in the upper left. In Supplemental Fig. 1A, the video observation was derived from a melanoma B16-bearing C57BL/6 (B6) mouse. The flow velocity (initially traveling from left to right on the screen and marked with yellow arrows) increased following the administration of the saline bolus, and then decreased following the first phenylephrine dose, resulting in a transient period of stasis. The velocity decreased further following the second phenylephrine dose, which generated a reversal of flow (now traveling from right to left, starting at 16:22:40 in the video, and marked with yellow arrows) within visualized vessels (two larger branches marked with yellow arrows above each highlighted vessel). The bolus and phenylephrine controls showed similar results, albeit of decreased magnitude (videos not included). Of note, the darkly pigmented B16 tumor can be seen overlying and obscuring short sections of these two vessels, specifically in the right upper portion of the screen.

Supplemental Fig. 1B corresponds to the still photos shown in Fig. 2. Interestingly, the flow velocity within this vessel also transiently decreased and stopped (occurring at approximately 20:39:00), consistent with the observations in the functional vessels within B16-bearing mice (Supplemental Fig. 1A). Following this event, the flow velocity increased approximately 3 min following administration of the phenylephrine dose (results after 1st dose shown). The bolus and phenylephrine controls alone did not restore non-functional vessels, but did similarly alter velocities (videos not shown) within the functional vessels.

In the last video observation (Supplemental Fig. 1C), which used a 4T1 breast tumor as an example, functional tumor vessels experienced a significant decrease in velocity following the phenylephrine injections, again demonstrating transient reversal of flow (occurring at approximately 15:34:90) consistent with the B16 and CT26 example videos. Blood flow normalized in the last portion of the video approximately 3 min after the 2nd phenylephrine injection, consistent with the short half-life of phenylephrine and prior videos depicting different cell lines. Interestingly, results were consistent across three tumor types, including melanoma (B16), breast cancer (4T1), and colorectal cancer (CT26).

**Figure 3:**
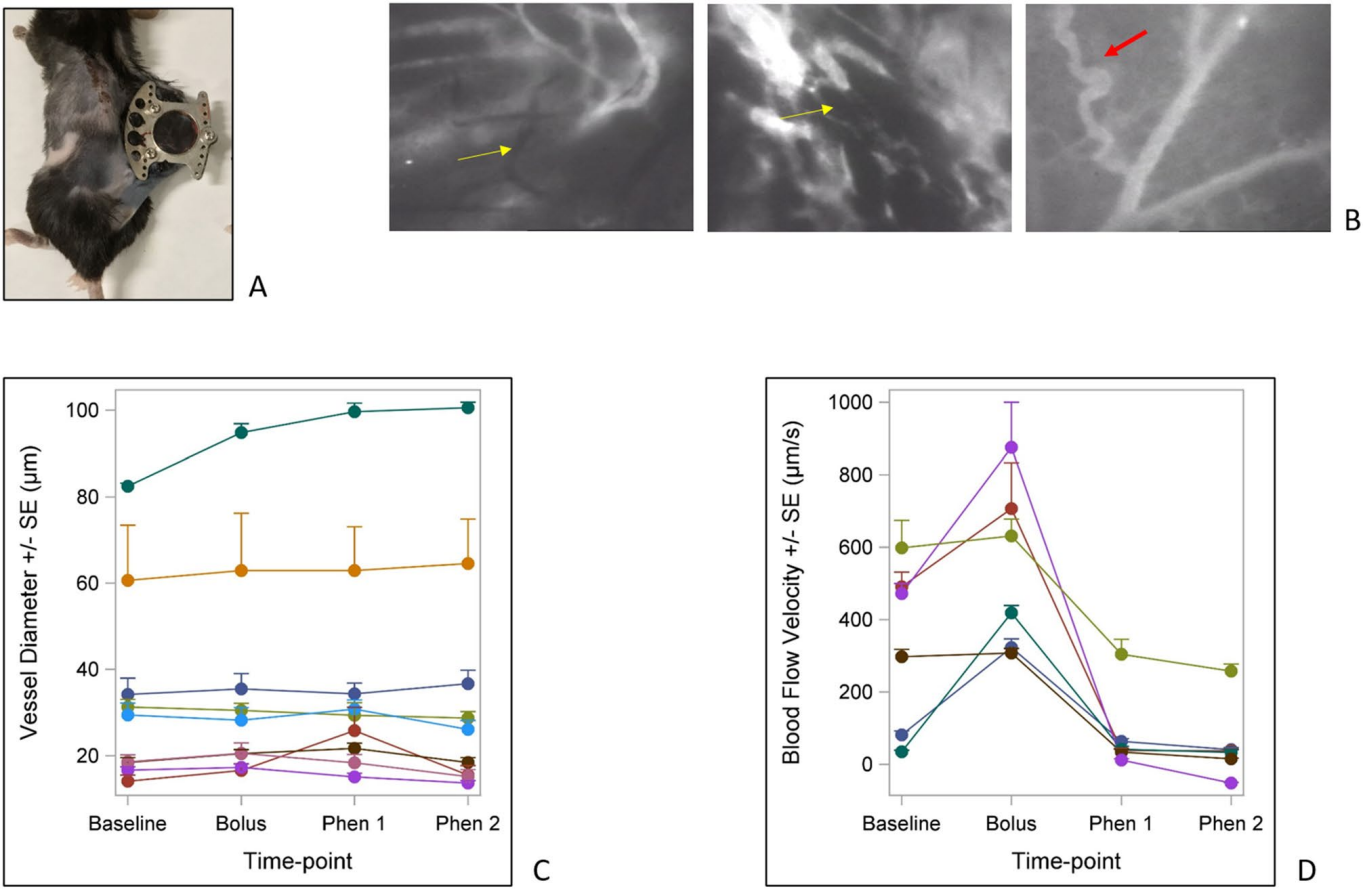
(**A**) Implanted window chamber in C57BL/6 mouse for IVM observation of B16 melanoma. (**B**) Aberrant characteristics of tumor-associated vessels. Yellow arrows highlight non-functional vessels. The red arrow highlights a tortuous, abnormal tumor vessel branching from a streamlined, normal appearing vessel. (**C**) Average B16 tumor-associated vessel diameters measured during dynamic control. There was no consistent change in vessel diameter. (**D**) Average B16 tumor-associated flow velocities during dynamic control. Velocities increased following the saline bolus and then decreased or even reversed following phenylephrine doses.

Figure 3 illustrates the gross B16-bearing window chamber (A), examples of the haphazard architecture of the tumor-associated vasculature (B), and summarizes the vessel diameter (C) and flow velocity changes during dynamic control (D), which were reproducible and quantifiable. Yellow arrows in (B) highlight non-functional vessels as characterized by the absence of fluorescent dye, and the red arrow highlights an aberrant, tortuous tumor vessel in juxtaposition to a normal appearing, streamlined vessel. These findings were common throughout IVM observations of each tumor type.

Figure 3C summarizes changes among the average cumulative vessel diameters for a specific IVM view for a given B16-bearing mouse (each line represents observations obtained from a single mouse). Overall, most vessel diameters remained consistent during the dynamic control protocol. Only one mouse showed an increase in diameters during the protocol, which had the highest baseline average vessel diameter. In contrast, the velocity changes during dynamic control showed a more consistent trend. Figure 3D reports the changes in velocity to observed vessels within separate B16-bearing mice. Velocity often increased following the saline bolus, and then decreased following the first phenylephrine dose with further decrease following the second phenylephrine dose. Some vessels demonstrated reversal of flow, as quantified by a negative value (line traveling below the 0 value on the y-axis), consistent with the real-time observations shown in Supplemental Fig. 1.

### Dynamic control of tumor vessels results in increased drug effect in extremity melanoma

Based on these effects of dynamic control on blood flow within tumor vessels, we hypothesized that dynamic control could enhance drug delivery to tumor and subsequently drug efficacy. We postulated that this would occur through a combination of restored flow through non-functional vessels and altered flow velocity through functional tumor vessels at the time of drug delivery. Restoration of peri-tumoral blood flow through non-functional vessels and a transient decrease in blood flow through functional vessels would be expected to result in an increased dwell time of the therapy (melphalan) within the target tumor, thereby producing increased exposure of the tumor to melphalan.

**Figure 4:**
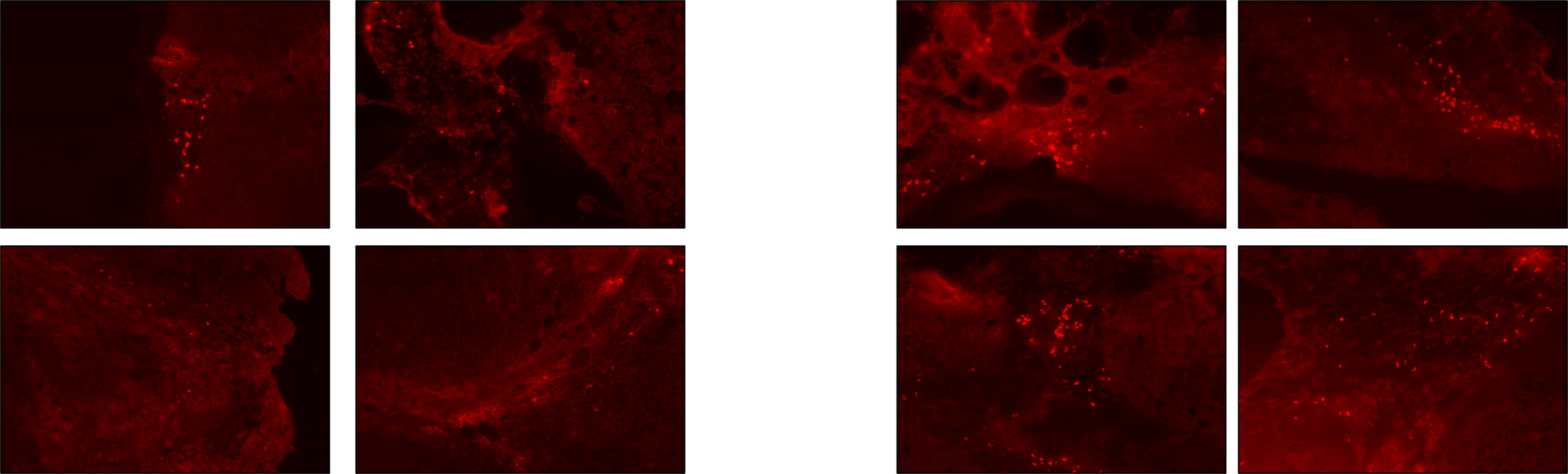
Fluorescent staining of DNA adducts. Left panel: B16 tumors treated with melphalan alone; Right panel: B16 tumors treated with melphalan plus dynamic control. Tumors were removed 2 days after treatment infusion. The number of DNA adducts was significantly higher for tumors treated with the combination of dynamic control and melphalan compared to melphalan alone (average number of observed DNA adducts per 20 × field: 120.5 versus 30.3, respectively, p = 0.029).

To test this hypothesis, we used an isolated limb perfusion mouse model of melanoma^23,26^. Dynamic control, in this case consisting of a systemic saline bolus plus phenylephrine limb infusion through the ILP circuit, increased the activity of melphalan. This was supported by the increased presence of DNA adducts in mice treated with melphalan plus dynamic control compared to mice treated with melphalan alone. Figure 4 shows that the number of DNA adducts was significantly higher for tumors treated with the combination of dynamic control and melphalan (right panel) compared to melphalan alone (left panel). The average number of observed DNA adducts per standardized 20 × field was 120.5 versus 30.3, respectively (p = 0.029), indicating an approximate fourfold increase in drug activity obtained with dynamic control. Control mice treated with vehicle only (saline) or dynamic control only (absence of melphalan) did not show any observable DNA adduct formation.

### Dynamic control of tumor vessels augments antitumor responses in a regional perfusion therapy model of melanoma and improves outcomes

**Figure 5:**
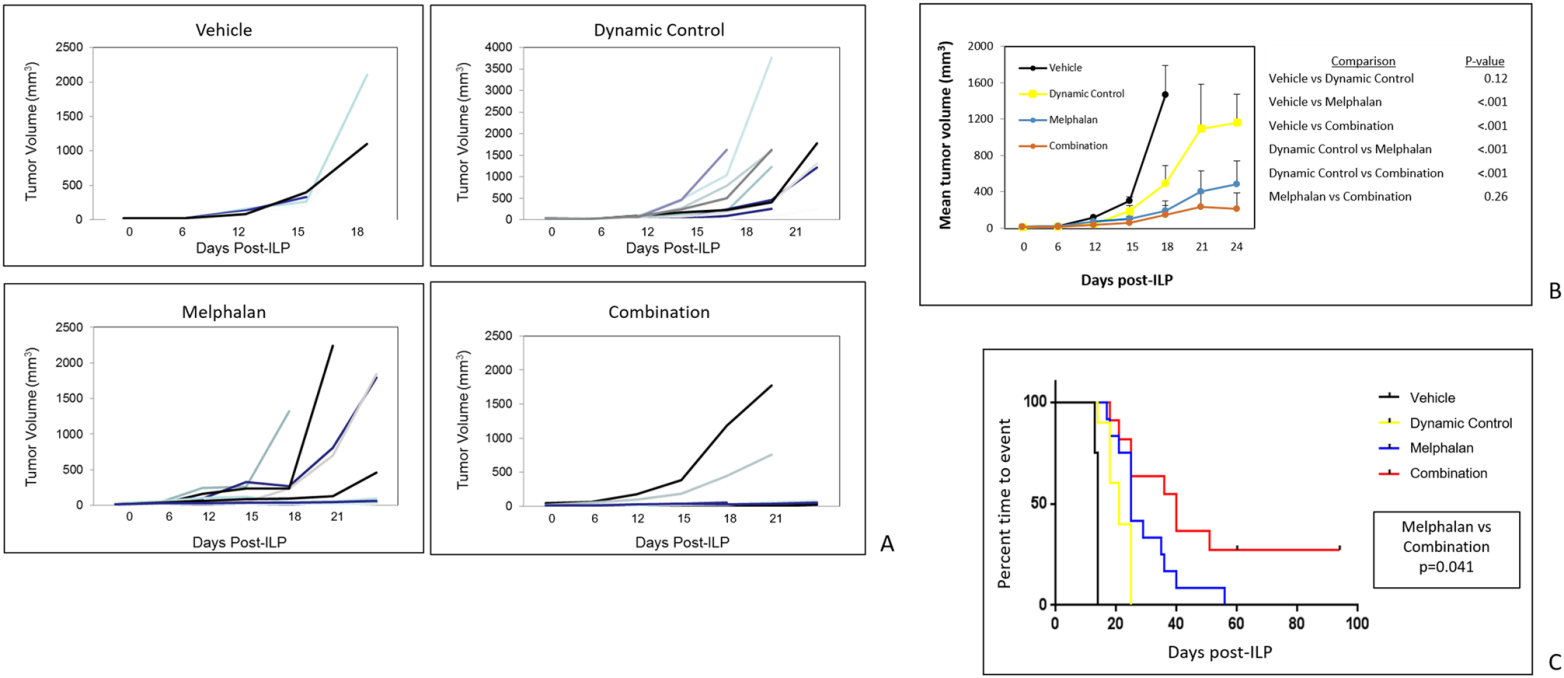
(**A**) Individual mouse B16 tumor volume curves among the treatment groups: vehicle (saline), dynamic control alone, melphalan alone, and the combination of dynamic control and melphalan. The combination group had the highest number of mice (10/12) with tumors under 50 cubic mm during the first 3 weeks after ILP, with the exception of two outlier mice. (**B**) Mean tumor volume curves among treatment groups. Error bars represent standard errors of the mean. The average tumor volume between the combination group and the melphalan alone group was not statistically significant, likely due to the presence of the two outliers in the combination group. (**C**) Kaplan–Meier curve showing statistically superior outcome for the combination group, even when compared to the melphalan alone group. There were 3 complete responses (25%) in the combination group, and no complete responses in any other group. Data were pooled from 3 replicate experiments.

Based on the finding that dynamic control increased the number of DNA adducts during regional limb perfusion with melphalan, tumor growth experiments were performed with the following groups undergoing ILP: (1) vehicle (saline) control, (2) dynamic control (saline plus phenylephrine) alone, (3) melphalan alone, and (4) dynamic control plus melphalan (combination). Figure 5 shows the tumor growth results and Kaplan–Meier curves. Time to event was defined as sacrifice due to endpoint tumor volume (2,000 mm^3^), sacrifice due to local symptoms as a result of the tumor, or death. Mice were pooled from three separate experiments (vehicle, n = 4; dynamic control alone, n = 12; melphalan alone, n = 12; combination, n = 12). Each replicate experiment had the same number of mice within each treatment group (dynamic control alone, melphalan alone, and combination; each n = 4). The first replicate also included a vehicle control (n = 4), whereas the second and third replicate did not include this negative control group. The main comparison of interest was between the melphalan alone group and the combination group.

Figure 5A shows the tumor growth results of each individual mouse within each treatment group. Because of two outliers in the combination group, the average tumor growth during the observed time period was not statistically significant between the melphalan alone and combination groups (p = 0.26), as shown in 5B. However, the Kaplan–Meier analysis did reach significance (p = 0.041) with the combination group showing the best long-term outcome with a 25% (3/12) complete response (cure) rate.

Overall, a low proportion of animals (6/40 or 15%) had events due to death. The majority of animals had events due to sacrifice when endpoint tumor volume was reached or when local symptoms were experienced, including tumor ulceration or impaired mobility. Specifically, the following proportions of mice were sacrificed based on these predefined endpoints: vehicle—3/4, dynamic control alone—10/12, melphalan alone—10/12, and combination—11/12. The 3 mice in the combination group that obtained complete response were euthanized 95 days after the ILP procedure.

### Combined treatment with dynamic control and melphalan does not result in increased toxicity

Recognizing that phenylephrine is a potent vasoconstrictor and could result in increased toxicity, we monitored for adverse events. There was no loss of limb or statistically significant increased skin reactions among the treatment groups (adverse event rate: vehicle—0/4; dynamic control—0/12; melphalan alone—1/12; combination—2/12). Toxicity among the 1 mouse in the melphalan alone group and the 2 mice in the combination group consisted of Wieberdink grade 3 superficial blistering of the lower extremity skin. No deep tissue layers were exposed, and these mice still maintained normal motor function.

**Figure 6:**
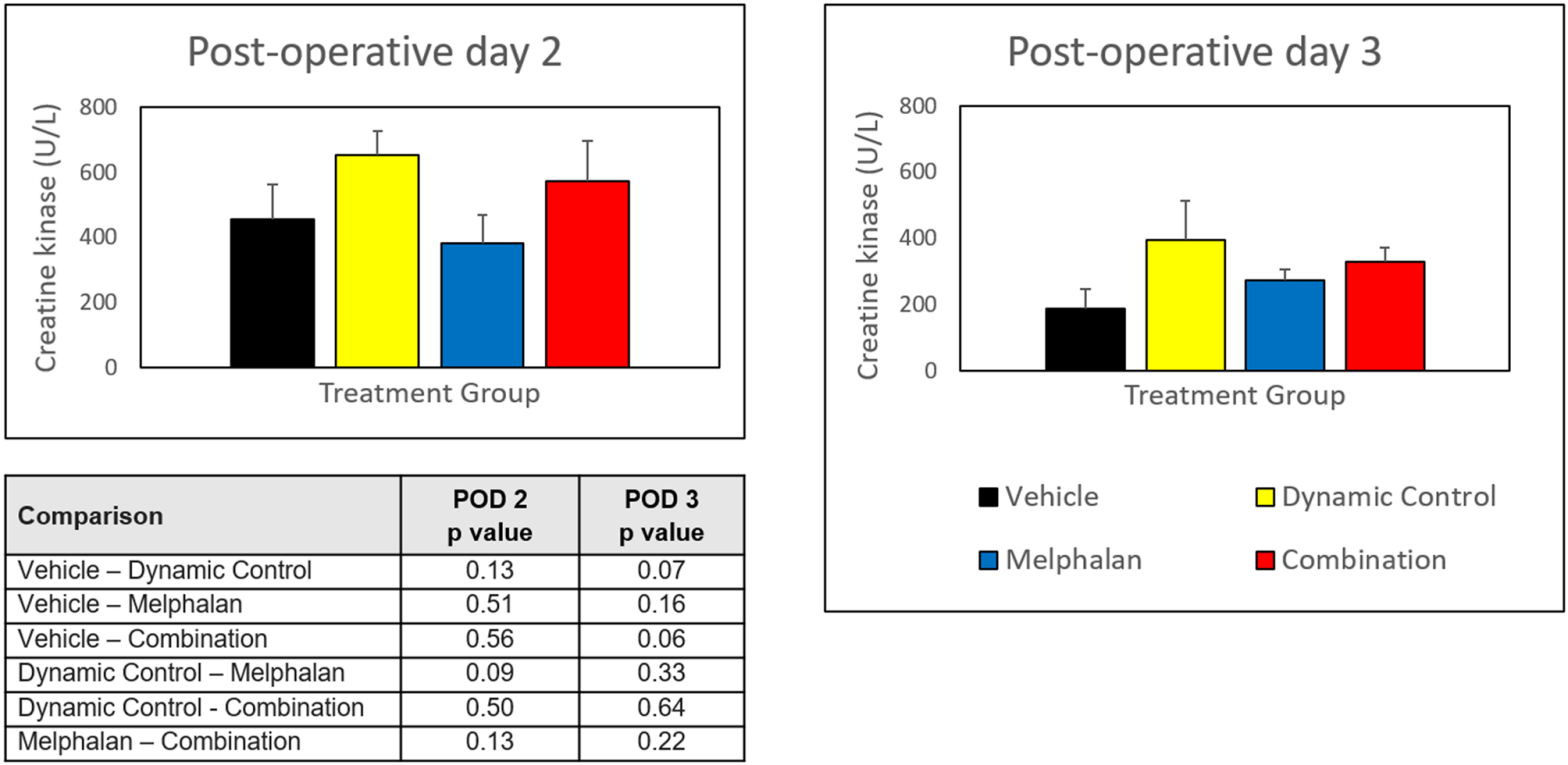
Creatine kinase (CK) levels following isolated limb perfusion on post-operative days 2 and 3. Error bars represent standard errors of the mean. No statistically significant differences were observed among the different treatment groups. In general, the CK levels decreased on post-operative day 3.

Measurement of serum creatine kinase (CK) levels post treatment showed no statistically significant differences among groups on post-operative day 2 or 3, when CK levels would be expected to peak and then drop, respectively (Fig. 6). There was a trend toward higher CK levels in mice receiving dynamic control (either alone or in combination with melphalan), though this was not statistically significant. There was also a general decrease in CK levels observed between post-operative days 2 and 3 among all groups.

## Discussion

In this study, the overall objective of improving antitumor responses was focused on the tumor vasculature as opposed to the tumor cells themselves. This innovative approach, termed dynamic control, restored blood flow through non-functional vessels and also transiently altered blood flow velocity in functional vessels, which were directly observed in real time using IVM. Although a decrease in flow velocity resulting in increased drug exposure to tumor may initially seem counterintuitive, the elevated interstitial pressure within tumors has actually been shown to (1) impede afferent blood flow into tumor and (2) facilitate efferent blood flow out of tumor^14,27,28^. Taken together, these two effects on blood flow limit the exposure or dwell time of treatment within the tumor itself^19,22,29^. Therefore, our study was built on the rationale that dynamic alterations to the tumor vasculature would result in optimal drug delivery, which was shown by demonstrating increased melphalan delivery and improved responses in a mouse melanoma model of regional chemotherapy perfusion.

This concept of tumor vessels acting as a functional barrier to systemic drug delivery has been previously described^13,14^. Treatment strategies that exploit the vascular interface between the tumor and the host, however, have been hindered by intrinsic characteristics of tumor vessels. For example, as shown in Fig. 3 and our previous work, tumor vessel architecture is often disorganized with a high proportion of non-functional areas, with up to 50% that lack detectable blood flow^30^. Furthermore, the elevated interstitial pressure generated within the tumor also inhibits perfusion to the tumor itself, both by limiting the inflow into the tumor and by facilitating the outflow from the tumor. Each of these aberrancies in tumor dynamics minimizes the amount of blood flow into the tumor and consequently has important implications on the delivery of anticancer treatment to target tumor cells. Cell-based immunotherapeutics are similarly dependent upon accessing the tumor via the vasculature, as improved outcomes have been directly correlated to immune cell infiltration within the tumor^24,25^. Limited T cell trafficking within tumor-associated vessels is widely accepted as a key mechanism of immune evasion^31–33^.

Because tumor vessels have been shown to express aberrant α receptors located on the vessel pericyte^13^, the phenylephrine in our dynamic control approach is likely acting on normal vessels outside of the tumor, increasing the efferent resistance to outflow of blood from tumor. This rationale would be consistent both with our studies using systemic injection of phenylephrine (Fig. 2 and Supplemental Fig. 1 showing the effects of systemic phenylephrine on melanoma implanted within dorsal skin window chambers) and our studies using regional perfusion with phenylephrine (Figs. 4 and 5 showing the effects of dynamic control on melanoma implanted into the hind limb). In either setting (systemic or regional), redistribution of intravascular volume from normal tissue to the tumor bed and increases in efferent resistance likely account for the observed dynamic changes in tumor blood flow.

While these results were the first to show the benefit of dynamic control to chemotherapy in a regional perfusion model, phenylephrine and other vasoactive agents have been shown to augment anticancer treatments in other settings^34–36^. Preclinical studies of intratumoral epinephrine and fluorouracil in a mouse sarcoma model reported superior responses with this combination compared to fluorouracil alone^37^. Similarly, the use of intratumoral phenylephrine in clinical trials has yielded improved responses for surface malignancies, including squamous cell cancer and other head/neck tumors^38,39^. These combination strategies have used phenylephrine in conjunction with intratumoral cisplatin to increase drug dwell time within the tumor as well as increase intratumoral hypoxia, both of which led to enhanced tumor responses. Other applications of vasoactive agents plus chemotherapy have shown promise, including their use in the local treatment of liver tumors. Vasoactive drugs including phenylephrine and angiotensin have been shown to redistribute blood flow to hepatic tumors and enhance the dwell time of radiolabeled therapies within the tumor tissue^40,41^. These findings were recapitulated in our mouse model of regional limb perfusion and were consistent with the rationale for our experiments. Interestingly, in addition to the effects of tumor vessel dynamics, vasoactive agents have also been shown to have possible anti-proliferative effects by acting through the α-1 receptor, which may also contribute to tumor responses^42,43^. In fact, several signaling pathways that could be affected by phenylephrine have been implicated in these studies, including cAMP, protein kinase C (PKC), and Extracellular Signal-regulated Kinase-1 (ERK1).

Current regional therapies for IT melanoma offer reasonable response rates in select patients, though the durability of response is often limited. Novel approaches to ILP include the addition of other agents to melphalan, such as actinomycin D (dactinomycin), tumor necrosis factor alpha (TNF-α), and bevacizumab^44,45^. While these combination approaches to regional therapies have shown improvements to outcomes in preclinical models, the added clinical benefits have been modest^46^. Combination of ILP and ILI with newer immunotherapies, including the anti-CTLA4 antibody ipilimumab and the anti-PD1 antibody pembrolizumab, are currently being investigated, but these are still far from becoming standard therapy^47^. In order to improve the durable outcomes of patients with IT melanoma, novel treatment strategies are needed.

**Figure 7:**
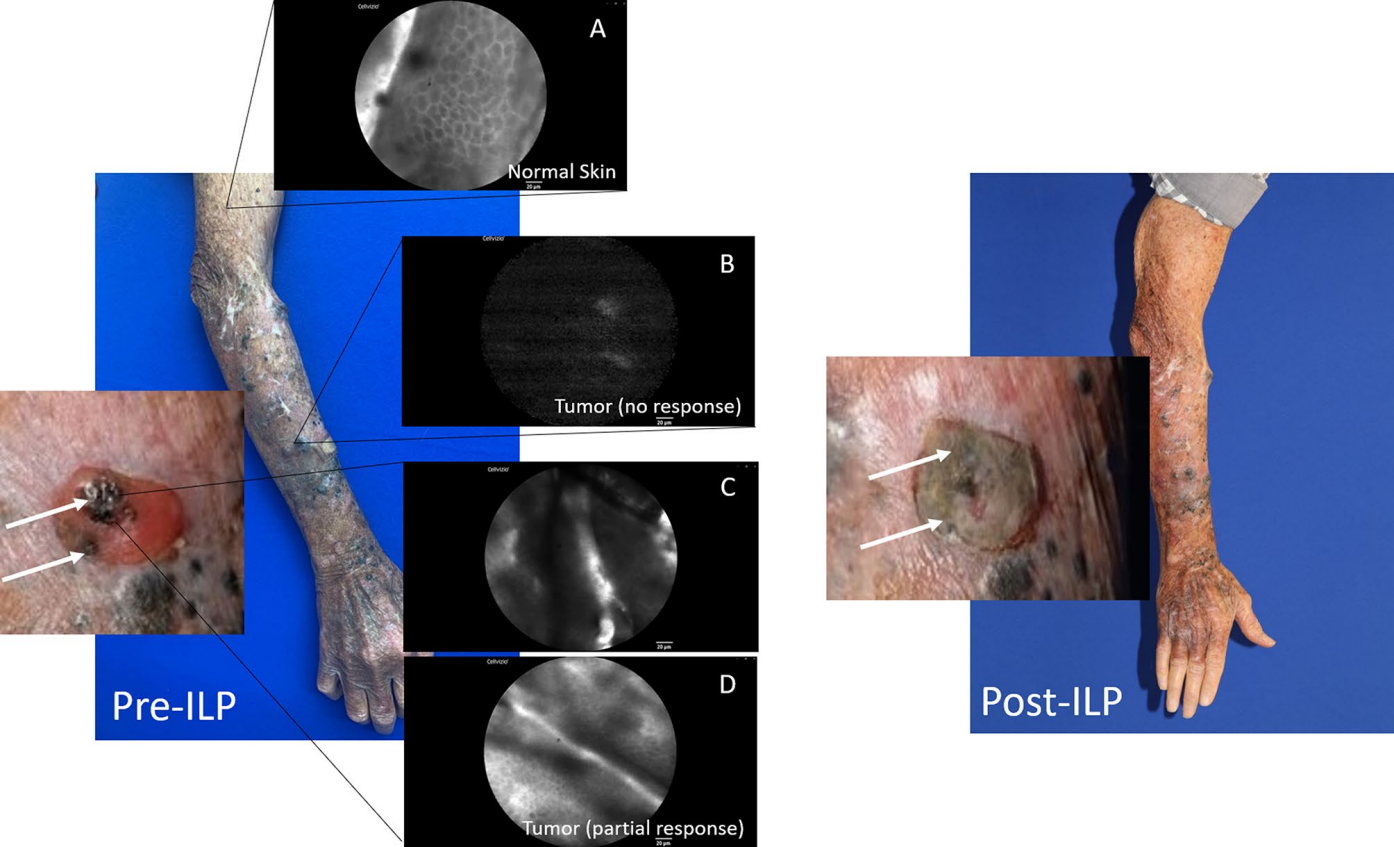
Human IVM in a patient with in-transit (IT) melanoma of the right upper extremity prior to undergoing ILP with melphalan. Insets (pre-ILP) show fluorescein uptake in normal skin (**A**), little to no fluorescein uptake in larger IT nodules that had no response (**B**), and mixed fluorescein uptake in smaller IT nodules that had partial response (**C**, **D**). White arrows (magnified views) indicate IT nodules that demonstrated fluorescein uptake and showed tumor response following ILP with melphalan.

To this end, our findings will form the basis for a clinical trial that will test our protocol for dynamic control of tumor vasculature in patients with IT melanoma who are candidates for regional therapy. Our group has developed an IVM microscope capable of performing real-time observations in human subjects^30^. This technology allows for the translational application of our findings and is the subject of ongoing clinical trials evaluating the feasibility of human IVM^48^. These include IVM in sentinel lymph node biopsy for patients with melanoma (NCT02857374), for patients with peritoneal carcinomatosis (NCT03517852 and NCT03297489), and for patients with solid tumors, including melanoma IT disease (NCT03823144)^49–52^. In fact, use of human IVM has shown that patients with melanoma often lack functional peri-tumoral vessels^30^, suggesting that systemically delivered therapies may not reach target tumor. As a recent example, one subject with IT melanoma enrolled in NCT03823144 (Intravital Microscopy in Human Solid Tumors) was shown to have variable proportions of non-functional vessels in different areas of the affected limb. This patient had multiple IT nodules afflicting the right upper extremity. In addition, he had several areas of cutaneous scars from both prior surgical excisions as well as previous unrelated trauma. Human IVM showed variable levels of fluorescein uptake in areas of normal skin, scar tissue, and the IT nodules (Fig. 7). Interestingly, larger IT nodules (≥ 1 cm) showed poorer perfusion compared to smaller IT nodules (< 5 mm). The larger nodules showed no treatment effect, whereas some of the smaller IT nodules did show partial response. While these findings represent only one subject from this study, these results are promising in that they appear to correlate with our previous findings ^30^. These observations also reflect the rationale behind dynamic vessel control as a potential means to improve drug delivery and efficacy in humans.

Because dynamic control targets tumor vasculature, this technique may easily be applied to other cancer types and potentially be given systemically in combination with other treatments, including chemotherapy and immunotherapy. Other cancer histologies, including colorectal cancer, ovarian cancer, sarcoma, and primary peritoneal mesothelioma, are also being investigated through clinical trials with IVM^50–52^. Interestingly, control of tumor vasculature may also affect the results of some radiotherapy treatments (i.e. radiofrequency ablation) by altering the blood flow and oxygen content during the treatment in order to generate or maximize a hypoxic environment^53^.

Our results suggest that there was no added toxicity by adding dynamic control to melphalan. The administration of systemic vasoconstrictors has been performed for patients undergoing ILI/ILP to regulate hemodynamics during the procedure and has been shown to be safe^54^. While there are no reports of regional administration of vasoconstrictors to patients during ILI/ILP, it may be extrapolated that the safety observed with systemic delivery of phenylephrine may be translated to regional administration. Furthermore, in our preclinical model, the perfusion was not heated but rather delivered at room temperature in order to minimize toxicity. However, our model of dynamic control with ILP at room temperature still generated responses. The use of papaverine, a potent vasodilator, is sometimes used clinically during ILI/ILP. However, papaverine has been shown to result in increased toxicity compared to those who do not receive it^55,56^. Whether the vasoconstrictor phenylephrine may add clinical toxicity during ILI/ILP similar to papaverine and if this would be impacted by heated therapy will be addressed in our next clinical trial.

We recognize that there are limitations to our study. The mechanism by which dynamic control functions was not addressed by this study. We hypothesize that dynamic control likely acts by shunting blood to the tumor bed through vasoconstriction of normal (non-tumor) peripheral and central blood vessels because tumor-associated vessels have been shown to have altered expression of α receptors. Our results suggest that there is a critical tumor mass after which dynamic control will no longer augment systemic chemotherapy. This may be the case because tumor growth kinetics outpace treatment effect. While addressing the tumor vasculature as a barrier to drug delivery is important, the therapy itself must also be effective at the cellular and/or molecular level once it is delivered. Thus, dynamic control must be performed as part of a multimodal approach to cancer treatment. Our study also tested dynamic control in a regional model of IT melanoma using isolated limb perfusion. It is unclear whether this approach will be similarly effective in models of distant metastases (for example metastatic melanoma to lung or liver). In addition, it is unknown whether dynamic control will be effective in combination with other drugs for different tumor histologies as tumors other than melanoma may likely affect the tumor-associated vasculature in different ways. It is also unknown how dynamic control with influence cell-based therapeutics such as adoptive T cell transfer or Car-T cell therapy. Lastly, while we are performing early translational studies of human IVM that show the feasibility of IVM in observing human tumor-associated vessels (including human IT disease), there are many considerations and steps needed to safely employ a strategy of dynamic vessel control in humans. For example, there are known contraindications to systemic phenylephrine, such as significant cardiovascular or cerebrovascular disease, that would preclude administration of vasoactive agents to these patients. In addition, ILP in humans is performed with hyperthermia. Whereas these results did not show increased toxicity in our mouse model of ILP, these studies were performed under normothermic conditions. Thus, dynamic control combined with melphalan during ILP in the treatment of human subjects may result in increased toxicity. These limitations and new questions will be addressed in future experiments and human trials.

In conclusion, we provide the first evidence that dynamic control of tumor vessels was feasible and directly observable via IVM using our protocol of volume expansion (saline bolus) plus phenylephrine. This approach to targeting the tumor vasculature as a means to enhance dug delivery and efficacy was supported by the increased activity of melphalan (through detection of DNA adducts) and antitumor responses (as shown by delayed tumor growth and superior time to event outcome). As tumor flow dynamics are likely to impact lymphocyte trafficking and extravasation into tumor as it pertains to vessel wall shear, future studies will also investigate the effect of our approach on cell-based immunotherapies, which are particularly relevant to melanoma and are being increasingly studied in other cancers. Lastly, the translational efficacy of these preclinical results will be the objective of our future trials utilizing human IVM and dynamic control.

## Methods

### Chemicals and reagents

Melphalan and phenylephrine reagents were purchased from Sigma-Aldrich and stored at 4 °C. Melphalan was dissolved in phosphate buffered saline (PBS) containing 1% dimethyl sulfoxide (DMSO) and 0.1% hydrochloric acid (HCl). Phenylephrine was dissolved in PBS.

### Tumor cells

B16 (subclone F10) melanoma cells (previously tested and authenticated) were obtained from ATCC (2006). Cells were cultured in RPMI 1,640 (Roswell Park Memorial Institute Medium) supplemented with 10% FCS (fetal calf serum), 2 mM l-glutamine, 100 U/ml penicillin, 50 μg/ml streptomycin, and 50 μM β-mercaptoethanol (Invitrogen). For additional IVM analyses, CT26 colon cancer cells and 4T1 triple negative (ER-/PR-/HER2-) breast cancer cells were similarly purchased, authenticated, and maintained.

### Mice

Female C57BL/6 mice (6–8 weeks old) were purchased from the National Cancer Institute (NCI) Mouse Repository (Frederick, MD). For the purposes of this study, male mice were not used as the primary comparison of interest was between the melphalan alone versus combination (melphalan plus dynamic control) groups. In addition, previous studies have shown no significant sex-based differences in B16 growth^57^. Prior to use, mice were allowed to acclimate to the animal facility for 1 week.

The technique for window chamber implantation has previously been described by our group^24,48^. Mice that had window chambers implanted were housed in rat cages to allow for better unobstructed mobility within the rat cage, which can be encountered in mouse cages (which have smaller dimensions). For additional IVM analyses with CT26 or 4T1 tumor cells, female BALB/c mice (6–8 weeks old) were used and also purchased from the National Cancer Institute (NCI) Mouse Repository (Frederick, MD).

### Intravital microscopy (IVM)

Methods for IVM have previously been described by our group^24^. Briefly, 10^5^ tumor cells suspended in PBS were injected into surgically implanted dorsal skin flap single-sided window chambers (Research Instruments, Inc.)^58^. For imaging of the tumor microvasculature, animals were anesthetized with isoflurane (1.5% with oxygen) and maintained at body temperature. Imaging began when tumors reached 5 mm in diameter, which occurred approximately 7–10 days after inoculation. All images were collected with a 2.5 × objective lens. Fluorescein isothiocyanate–dextran (FITC-dex) given systemically via tail vein injection was used as the fluorescent dye to enhance the quality of the observations.

### Dynamic control protocol for IVM observation of tumor-associated vessels

Figure 1 shows the schematic outlining the dynamic control protocol for IVM observation. Baseline observations before and after the administration of FITC-dex were performed with the goal of obtaining 1–2 fields of view harboring tumor vessels. Following baseline observations for 3–5 min, a 500 µl saline bolus was given systemically via tail vein injection followed by a 3-min observation period. This was followed with the first systemic injection of phenylephrine (10 µg/mouse) suspended in 50 µl PBS, followed by a 3-min observation period of the initial fields of view. Phenylephrine dosing was obtained from prior studies^40,59^, as well as through testing several dose-responses, including 1, 5, 10, 20, and 50 µg/mouse. The10 µg/mouse dose achieved the observable and quantifiable changes reported in the Results and minimized toxicities on the mice, with the main side effect being arrythmia. Phenylephrine has a rapid onset of action of approximately 1–2 min. Lastly, a second systemic injection of phenylephrine (10 µg/mouse) was given followed by a 5-min observation period. Mice were euthanized at the end of the procedure. Mice that received saline bolus alone or phenylephrine alone were used as controls.

### Post hoc IVM analyses

As per our previous studies^24,30^, vessels were defined morphologically, and a single vessel was described as beginning at a branch point continuing to the next branch point. To be measured, vessels had to be 100 µm in length with no branch points. ImageJ software was used to measure vessel diameter (D) at the vessel’s largest width. Blood flow velocity (v) was evaluated by determining the time that distinct features (i.e. aggregates of intravascular cells) in the fluorescent dye (FITC-dex) would take to travel a measurable distance and then averaging measured distances for at least 3 points per vessel. Non-functional vessels were defined as vessels which did not have any uptake of FITC-dex or discernable blood flow.

### Tumor establishment for ILP model

Female B6 mice were injected subcutaneously in the lateral aspect of the left distal hind limb with B16 melanoma cells. The cells (1 × 10^5^ per injection) were delivered in 0.1 mL of PBS. Treatments initiated when tumors reached 5 mm in their longest dimension (approximately 7 days post-inoculation).

### Treatment of B16 tumor–bearing mice

The mouse ILP procedure and melphalan dosing that recapitulates clinical parameters was employed as previously described by our group and others^23,26^. Targeted drug delivery has been shown to occur without leak into the systemic circulation. The ILP flow rate was 0.15 ml/min (PBS) for 20 min. Mice were treated according to the following groups: (1) vehicle (saline) control, (2) dynamic control alone (saline bolus plus phenylephrine at the dose of 10 µg/mouse), (3) melphalan alone (20 µg/ml), and (4) combination (dynamic control plus melphalan). In this ILP model, the saline bolus (500 µl) was administered systemically followed by the limb perfusion containing phenylephrine (10 µg). Treatments were performed as a single dose in the continuous, one-time perfusion for the 20-min period. Perfusion was performed at room temperature via the superficial femoral artery (without a tourniquet or collection of venous drainage as in ILP). We intentionally decided to perform the perfusion at room temperature in order to minimize the toxicity that was expected to occur from the combination of heated therapy (42 °C) and vasoconstriction caused by phenylephrine.

To minimize bias among groups based on the initial tumor size and heterogeneous tumor growth kinetics, mice were randomly assigned to treatment groups so that each cohort had a similar distribution of starting tumor sizes. Time to event was defined as sacrifice when mice reached the endpoint tumor volume (2,000 mm^3^), sacrifice due to local symptoms as a result of the tumor (such as tumor ulceration or impaired mobility), or death. Mice were pooled from three experiments (vehicle, n = 4; dynamic control alone, n = 12; melphalan alone, n = 12; combination, n = 12). The three experiments were replicates that included the same numbers of dynamic control alone (n = 4), melphalan alone (n = 4), and combination (n = 4). The first experiment included the vehicle control (n = 4). In the second and third experiments, the vehicle control was not included (n = 0). This was decided a priori based on the known tumor growth kinetics in the vehicle (negative) control group^26^, no expected effects of the vehicle (saline) on B16 tumor growth, and the primary comparison of interest being melphalan alone versus the combination of melphalan and dynamic control.

### Fluorescence immunohistochemistry

In separate experiments, excised B16 tumors from 20 ILP treated female mice (n = 5, vehicle; n = 5, dynamic control alone, n = 5, melphalan alone; and n = 5, combination) were flash-frozen using Optimal Cutting Temperature (OCT) compound (Tissue Tek) that was submerged in liquid nitrogen. They were then cut into 9 μm cross-sections using a cryotome (Thermo Scientific) and maintained at − 24 °C to − 28 °C. Sections were then fixed in methanol-acetone for 30 min and then blocked for 10 min at room temperature with 5% goat serum (Jackson ImmunoResearch) in PBS.

The mechanism of action of melphalan generates DNA adducts, which can be detected using the antibody MP5/73^23^. This antibody was utilized to quantify the activity of melphalan within tumor cells. Sections were incubated with primary antibody (1:50 rat anti-mouse MP5/37, BD bioscience) at room temperature for 1 h, followed by a 30-min incubation with a goat anti-rat rhodamine-conjugated (red fluorescence) secondary antibody (1:100; Jackson ImmunoResearch) at room temperature. Samples were rinsed with PBS 3 times for 5 min each between every consecutive step. MP5/73 + cells (Apoptag Kit; Millipore) were quantified in at least 10 fields (average unit area of each field, 0.34 mm^2^) of nonsequential 9-μm-thick cryosections. Images of whole tumors were recorded at × 20 magnification and subsequently segmented and evaluated in ImageJ and Adobe Photoshop. Distinct immunoreactive tumor cells were counted. Mean numbers of MP5/73 + cells were calculated among the different treatment groups.

### Assessment of toxicity

Established local toxicity grades were assigned to each perfused limb. Reactions were evaluated daily, and the greatest toxicity observed was recorded. Morbidity was measured via a modified Wieberdink scale^60^. Serum creatine kinase (CK) levels (U/L) served as a marker of muscle toxicity. Blood (~ 50 µl) was obtained from each mouse 48 and 72 h after treatment using the superficial temporal vein. CK levels were measured in serum samples using a VITROS 5.1 FS instrument (Ortho Clinical Diagnostics, Inc.).

### Assessment of tumor responses

Three perpendicular axes of the tumors were measured approximately every other day using external digital calipers (Control Co.). Tumor volume was calculated using the formula 1/2 × length × width × height. Mice that died or were euthanized due to morbidity, tumor ulceration, or tumor reaching the size endpoint (2,000 mm^3^) were classified as events.

### Statistical analyses

The therapeutic indices of tumor volumes, serum CK levels, and MP5/73 staining data were each assessed by a two-tailed Student t test. Comparisons among the serum CK levels were calculated using the Holm-Bonferroni adjustment). The association between velocities and treatment time-points (i.e. saline bolus, 1st phenylephrine dose, and 2nd phenylephrine dose) within each vessel were evaluated using one-way ANOVA models, with Tukey adjust post-hoc comparisons. A linear mixed model was used to account for the inherent correlation between observations from the same vessel within a given mouse. All model assumptions were verified using the appropriate diagnostic plots. Tumor growth was assessed by ANOVA (GraphPad Prism, Version 6 software, GraphPad Software, Inc.). Error bars represent standard errors of the mean unless otherwise noted. Standard Kaplan–Meier methods were used to perform the time to event analysis. Values of p < 0.05 were considered significant.

### Study approval

Animal protocols were approved by the IACUC of both the Roswell Park Comprehensive Cancer Center (IACUC protocol #1153ML: Analysis of lymphocyte trafficking in murine adoptive immunotherapy protocols) and Mayo Clinic Florida (IACUC protocol #A00003049-17: Effects of tumor vessel control on systemic anti-cancer therapies). All methods described above were carried out in accordance with all relevant institutional guidelines and regulations.

## Supplementary information

Supplementary Information 1.

Supplementary Information 2.

Supplementary Information 3.

Supplementary Information 4.

## Abbreviations

ANOVA: Analysis of variance
cAMP: Cyclic adenosine monophosphate
CK: Creatine kinase
CTLA4: Cytotoxic T-Lymphocyte Associated Protein 4
DMSO: Dimethyl sulfoxide
ERK: Extracellular Signal-regulated Kinase-1
FITC: Fluorescein isothiocyanate
HCl: Hydrochloric acid
ILI: Isolated limb infusion
ILP: Isolated limb perfusion
IT: In-transit
IVM: Intravital microscopy
LVI: Lymphovascular invasion
OCT: Optimum cutting temperature
PBS: Phosphate-buffered saline
PKC: Protein kinase C
RPMI: Roswell Park Memorial Institute medium
TNFα: Tumor necrosis factor alpha
VEGF: Vascular endothelial growth factor
