# Supplementary material for: Dynamic control of tumor vasculature improves antitumor responses in a regional model of melanoma
Source: Sci Rep. 2020 Aug 6;10:13245. doi: 10.1038/s41598-020-70233-5 (PMC7413248; doi:10.1038/s41598-020-70233-5)
Supplement: Supplementary file 4 — Supplementary Information 4. [file 41598_2020_70233_MOESM4_ESM.docx]

SUPPLEMENTARY MATERIAL for:

**Dynamic control of tumor vasculature improves antitumor responses in a regional model of melanoma**

*Running Head*: Effects of tumor vessel control

Emmanuel M. Gabriel MD, PhD, FACS^1^, Minhyung Kim MD^2^, Daniel T. Fisher PhD^2^, Colin Powers MD^2^, Kristopher Attwood PhD^3^, Sanjay P. Bagaria MD^1^, Keith L. Knutson^4^, and Joseph J. Skitzki MD^5^

1. Department of Surgery, Section of Surgical Oncology, Mayo Clinic, Jacksonville, FL

2. Department of Immunology, Roswell Park Comprehensive Cancer Center, Buffalo, NY

3. Department of Biostatistics, Roswell Park Comprehensive Cancer Center, Buffalo, NY

4. Department of Immunology, Mayo Clinic, Jacksonville, FL

5. Department of Surgical Oncology, Roswell Park Comprehensive Cancer Center, Buffalo, NY

Corresponding Author:

Emmanuel M. Gabriel, MD, PhD, FACS

Assistant Professor of Surgery

Department of Surgery

Section of Surgical Oncology

Mayo Clinic Florida

4500 San Pablo Road

Jacksonville, FL 32224

Telephone: 904-953-2523

Email: Gabriel.Emmanuel@mayo.edu

**Supplemental Figure Legend**

Supplemental Figure 1. Intravital microscopy videos depicting real-time, directly observable changes to tumor vasculature following the dynamic control protocol. (A) shows a representative video of a B16 melanoma-bearing B6 mice. An increase in flow velocity following the saline bolus was noted (with blood flow initially traveling in the left to right direction), followed by a marked decrease in velocity, a transient period of static flow, and then a reversal of flow velocity following phenylephrine administration (vessels marked with yellow arrows showing blood flow now traveling in the reverse, or right to left, direction). Darkly pigmented B16 melanoma cells can be seen overlying portions of the highlighted vessels, mostly in the right upper quadrant of the field of view. (B) shows a representative example of a CT26 colon cancer-bearing mouse pertaining to the still images in Figure 2. This video highlights restoration of flow through an initially nonfunctional vessel (characterized by the lack of fluorescent dye uptake and no observable flow), which was achieved with dynamic control. Lastly, (C) shows a representative example of a 4T1 breast cancer-bearing mouse, which displayed similar effects of dynamic control to the B16 and CT26 mice.
